# Influence of Riverbed Incision and Hydrological Evolution on Water Quality and Water Age Based on Numerical Simulation: A Case Study of the Minjiang Estuary

**DOI:** 10.3390/ijerph18116138

**Published:** 2021-06-06

**Authors:** Peng Zhang, Lanyimin Li, Yishu Wang, Chengchun Shi, Chenchen Fan

**Affiliations:** 1 School of Environmental and Municipal Engineering, North China University of Water Resources and Electric Power, 136 Jinshui East Road, Zhengzhou 450046, China; fancc2019@163.com; 2 Shanghai Municipal Engineering Group, Shanghai Municipal Engineering Design Institute, 901 North Zhongshan Road, Shanghai 200092, China; lanyimin_li@163.com; 3 South China Institute of Environmental Sciences, China Ministry of Ecology and Environment, 16 Ruihe Road, Guangzhou 510530, China; wangyishu@scies.org; 4 Fujian Research Academy of Environmental Sciences, 10 Huanbeisan Village, Fuzhou 350013, China; stonerainman@126.com

**Keywords:** Minjiang River Estuary, low dissolved oxygen, water age, water quality, riverbed incision

## Abstract

In recent years, problems such as water quality deterioration, saltwater invasion, and low oxygen have appeared in estuaries all over the world. The Minjiang River in Fujian, as a typical tidal estuary area, is facing these thorny problems. In this paper, the effects of topography and hydrologic evolution on the water age and water quality of the lower reaches of the Minjiang River were simulated by building a hydrodynamic and water quality model. The results show that: (1) It was found that the riverbed incision of the lower reaches of the Minjiang River led to the overall decline of river water level, the increase of river volume, and the increase of downstream water age, which eventually led to the decrease of dissolved oxygen (DO) and the deterioration of water quality in the downstream from Shuikou to Baiyantan. However, the decline of topography led to the increase of tidal volume in the estuary, the enhancement of the dilution effect of oxygen-rich water bodies in the open sea, and the increase of DO in the lower reaches of Baiyantan. (2) Under no tidal action, the concentration of pollutants in the water of the North Channel increased, the DO decreased, and the DO decreased from Baiyantan to the offshore water. After the enhancement of tidal action, the dilution of oxygen-enriched water from the offshore water increased, and the DO increased. (3) The hydrological and water quality characteristics of the upper part of the lower reaches of the Minjiang River were mainly controlled by topography, runoff, and pollutant discharge, which were more affected by the tidal current transport operation and pollutant discharge near the open sea. In recent decades, the deterioration of water quality and the aggravation of saltwater intrusion in the Minjiang River were closely related to the serious topographic downcutting. The results provide a scientific basis for revealing the deterioration of estuary water quality and long-term management of the estuary.

## 1. Introduction

In recent years, people are increasingly concerned about the estuary and coastal water environment. Estuaries, as heterotrophic ecosystems where large numbers of organic substance transported by rivers and inputted from the ocean are mineralized [1,2], have appeared low DO and deterioration of water quality around the world [3,4,5]. Estuarine discharge, topography (volume), tides, pollutant emissions etc., playing a role in controlling the estuarine environment [6,7]. Different from the water bodies of lakes or inland rivers, the migration and diffusion of estuary materials is a complex process, which is not only affected by runoff and tide, but also by gravity cycle, wind-driven and unique topography. The magnitude of the flow will affect the speed of nutrient transport out of the estuary, but also affect the degree of river stratification (gravitational circulation exchange). Spring-neap variations in tidal may result in tidally driven mixing, stratification, and gravitational circulation [3,8]. The width and depth of the estuary cause the difference of the volume and the tidal current of the river, tend to the transport and diffusion of pollutants in the river, and the gravitational circulation exchange [9,10].

In recent decades, with the increasing interference of human activities on the estuary system, the evolution of the estuary delta and the geomorphologic evolution of the estuary channel have been significantly affected [11]. For example, the impact of reservoir dam construction on the estuary geomorphologic evolution caused by more and more attention due to the sharp decrease of sediment inflow in the basin, channel dredging, and artificial sand mining [12,13]. Dam construction in the global river basin has a profound influence on water and sediment transportation [14]. Walling and Probst [15] believed that about 40% of the global river runoff and 25% of the sediment were intercepted behind the dam. Channel dredging and artificial sand mining will affect the changes of boundary conditions and local hydrodynamic forces of the channel, resulting in corresponding changes in the evolution process of channel topography in the estuary. Yao et al. [16] studied the waterway maintenance, and a new waterway excavation project in Lingdingyang Bay has a significant impact on underwater topography and beach evolution, resulting in the river topography the shallower the shoal, the deeper the deep channel. The dredging of the downstream channel of the Minjiang River and sand mining of the channel have led to serious riverbed cutting, changes in the hydrodynamic conditions of the South Channel and the North Channel, and threats to the safety of the embankment [17].

To better understand the changing process of hydrodynamics and water quality in estuary under different influencing factors to improve the health of the aquatic system, it is of great significance to provide suitable time scales for representing mass exchange and transport processes spatially, which can be applied to determine the biogeochemical process and hydrodynamic process. Many researchers [18,19] introduced time scales to quantify the exchange and transport processes and to assess the assimilative capacity of a water body. These time scales include water age, transit time, resident time, turnover time, flushing time, etc., [20,21]. Different time scales have different definitions and expressions according to different authors. Bolin and Rodhe (1973) summarized previous results and introduced a more rigorous definition for the time scales of ‘age’, ‘transit time’, and ‘turnover time’. Because time scales are spatial and temporal variations in a large estuary, water age and flushing time have been extensively used to study time scales and nutrient budgets in estuaries [22]. Water age defined in this study is the time elapsed since the water parcel under consideration exited the region in which its age is prescribed to be zero, or particularly, the time elapsed since a water particle is discharged from the headwater of an estuary [23]. The aged concept of a water parcel has been used as a timescale to quantify pollutant transport in lagoons, estuaries, and oceans [7,24]. Flushing time of estuaries is commonly defined as the time needed to replace the freshwater already in the estuary (freshwater volume) at the rate of freshwater inflow, which expresses the ratio of freshwater volume over the freshwater flow rate, but water can never be “emptied” since estuaries constantly exchange with the sea. Therefore, another practical way to estimate estuarine flushing is to define the removal percentage, such as 50%, 75%, or 95% removal [25].

Because the number of pollutants is often associated with freshwater inflow and tidal mixing, we can use water age to analyze the pollutant transport or the overall characteristics of the estuary. For example, an estuary with short water age is unlikely to cause water bloom or heavy metal pollution because nutrients or toxic or harmful substances are quickly washed into the ocean. In this paper, based on the constructed hydrodynamic water quality model of the lower Minjiang River basin, we used the Minjiang Estuary as a case study to calculate a spatially varying mean age and water quality under different hydrological conditions and topographical conditions, and the effects of hydrological evolution and riverbed incision on the age and water quality of the Minjiang estuary are predicted and analysed. By using the numerical model to simulate the scene, the pollutant migration process and the cause of water quality deterioration can be well explained.

## 2. Materials and Methods

### 2.1. Study Area

**Figure 1:**
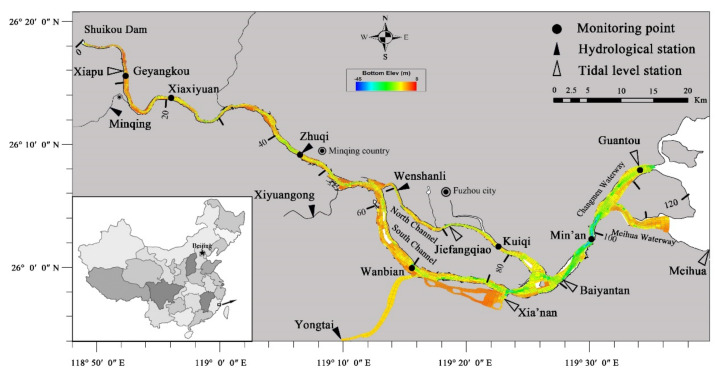
Model grid and topograohical sketch map unit: Luo-zero elevation (m).

**Figure 2:**
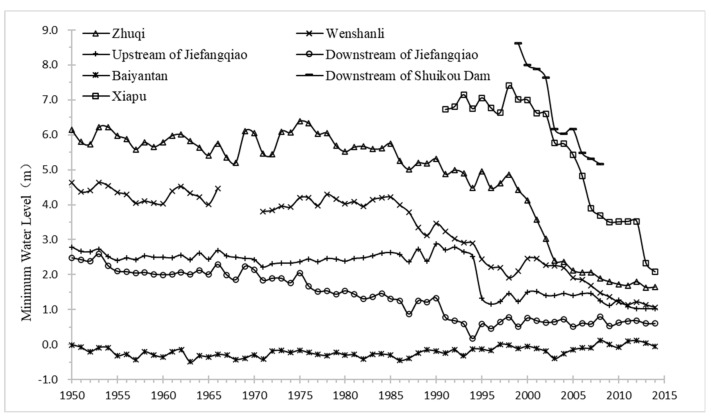
Annual lowest water level changes at representative stations in the lower reaches of Minjiang River.

The Min River is the largest single river flowing into the sea in Fujian Province, China (541 km) [17,26]. The lower reaches of the Minjiang River, with 117 km from the Shuikou Dam to coastal outfall, is located in the Minjiang watershed between 25°55′–26°18′ N and 118°48′–119°40′ E (Figure 1). The average annual flow of the Minjiang River Basin was 1760 m^3^ s^−1^, of which rainfall is the highest in summer and the smallest in autumn. Shuikou Reservoir is not completely seasonal adjustment in the dry season. However, there are still 1% of the daily average flows of Shuikou Hydropower Station lower than the minimum ecological flow (308 m^3^ s^−1^). The Minjiang River is divided into the South Channel and the North Channel by Nantai Island at Huaiantou and is merged at Mawei. Statistics on the lowest water level changes of hydrological stations and water level stations in the lower reaches of the Minjiang River from 1950 to 2014 are shown in Figure 2.

The Minjiang estuary is a strong tidal continental estuary, and the tidal type in the sea area within the Chuanshi Island is a regular semidiurnal tide. Two rises and two falls a day, with a tidal cycle of about 12 h and 25 min. The tide gradually deforms from the outer channel to the estuary, and the duration of the ebb tide increases, the duration of the flood tide shortens, and the tidal difference also gradually decreases.

### 2.2. Data Used

Topographic data and river section data of the lower reaches of the Minjiang River come from Fujian Port and Navigation Administration Survey Center (FPNASC). In addition, the latest chart information was supplemented near the sea, and the elevation is Luo-zero elevation. The outer boundaries of the model were set according to external conditions, and the flow and tide data (two maximum and minimum values in a day) was obtained from the Year Book of Hydrology P.R. CHINA (YBHC) in 2013. The water quality data onto the regular monitoring points of the Minjiang River come from FRAE [17]. Water samples were collected with Niskin bottles at 1 m below the surface, and the distribution of the monitoring points are shown in Figure 1. Data of automatic water quality monitoring station is every 4 h and routine monitoring data is monitored every month. Water quality monitoring is carried out according to China National Water Quality Standards (GB3838-2002). The concentration of TN, TP, NH_4_^+^, and DO were measured using an automatic water quality analyzer (AA3, SEAL, Ludwigshafen, Germany). In addition, the samples were taken to the laboratory, and BOD_5_ was determined by the diffusion and seeding method.

### 2.3. Model

#### 2.3.1. Description

The Environmental Fluid Dynamics Code (EFDC) was applied in the Minjiang River, including hydrodynamics, water ages, and water quality [17]. It was originally developed by the Virginia Institute of Marine Science as authorized by US EPA. EFDC has been successfully applied to a wide range of environmental studies simulating circulation, density or thermal stratification, sediment transport, eutrophication, and water quality in numerous lakes, rivers, wetlands, estuaries, and coastal regions [27,28,29].

#### 2.3.2. Configuration

In this paper, EFDC used orthogonal curvilinear coordinates, allowing grid sizes to vary and fit the estuary coastline (Figure 1). A total of 2743 grid cells existed in the study domain with grid size varying from 50 m to 1000 m (Figure 1) with three uniform vertical sigma layers. The actual cell size and shape resulted from grid optimization and orthogonality. Most importantly, EFDC included water age, sediment transport, and water quality. The 15 state variables in the water column were simulated by the water quality model, which contains green algae, three types of carbon (refractory particulate organic carbon, labile particulate organic carbon, dissolved organic carbon), five types of nitrogen (refractory particulate organic nitrogen, labile particulate organic nitrogen, dissolved organic nitrogen, NH_4_^+^, nitrate, and nitrite nitrogen), four types of phosphorus (refractory particulate organic phosphorous, labile particulate organic phosphorus, dissolved organic phosphorous, total phosphate), DO and chemical oxygen demand. The bottom topography data were obtained from FPNASC and interpolated into the model grids. The time step was set to 60 s to satisfy the Courant–Friedrich–Levy (CFL) criterion. The model was run on 1 December 2012. In order to make the output stable, the results were extracted from 0:00 on 1 January 2013.

##### Boundary Conditions

Atmospheric boundary conditions: mainly consider the wind field (wind speed and direction), air pressure, temperature, relative humidity, evaporation, rainfall, solar radiation and cloud cover, data from China Meteorological Network; daily rainfall and evaporation data from the YBHC in 2013.

Upper boundary conditions: the upper boundary of the model was the measured discharge of Shuikou Hydropower Station, and the daily temperature data of Xiapu hydrological station was used; water quality boundary was determined according to the conventional water quality monitoring section value.

Tributary boundary conditions: three tributaries of Meixi, Xiyuanxi, and Dazhangxi have hydrological stations, whose flows were provided by hydrological stations. The boundary conditions of other tributaries were obtained by analogy according to the catchment area of each tributary. The water quality boundary conditions of tributaries were generalized to the sub-basins where each tributary was located according to the calculated number of pollutants into the river.

Open sea boundary conditions: the tidal level, salinity, and temperature were provided by the hydrodynamic model of Shuikou Hydropower Station—Minjiang open sea (Mazu Island and Xiquan Island). The tidal level was calculated by the global tidal model TPXO 6.2 developed by Oregon State University. The harmonic results were verified by the measured data of the Huangqi hydrological station, and the average absolute error was within 0.1 m [30]. The downstream water quality boundary was determined according to the conventional water quality monitoring section value.

Due to the lack of parameter values of atmospheric settlement and sediment release, according to the relevant reference [31], the dry and wet settlement in the atmospheric boundary in this paper adopted the mean value, and the sediment release adopted the release value with an uneven spatial and temporal distribution.

According to the data of 2013 (environmental statistics, statistical yearbook, and key monitoring units), the total inflow of COD, NH_4_^+^, TN, and TP in the lower reaches of Minjiang River in 2013 were 102363 t y^−1^, 11267 t y^−1^, 21168 t y^−1^, and 1778 t y^−1^ respectively; The proportions of COD, NH_4_^+^, TN, and TP of point source were 82%, 81%, 74%, and 64% respectively.

##### Initial Conditions

River nutrients, DO, salinity, and temperature were set based on observed values from the measured results, and the water surface elevation were set at 2.5 m. The model was initially run for several days for each flow condition to obtain a dynamic equilibrium condition. The flow field, salinity, and water concentration distribution under this equilibrium condition were then used as the initial condition for the model calibration so that the model could be ‘hot’ started [24].

#### 2.3.3. Calibration and Validation

The data from 2012 and 2013 were selected to calibrate and validate the established model. The WQ model contains more than 100 parameters, and the calibration values of the main parameters were shown in Table 1. From the results of calibration and validation, it can be seen that the model could reasonably reflect the change process of DO.

##### Hydrodynamic Model Parameters

**Figure 3:**
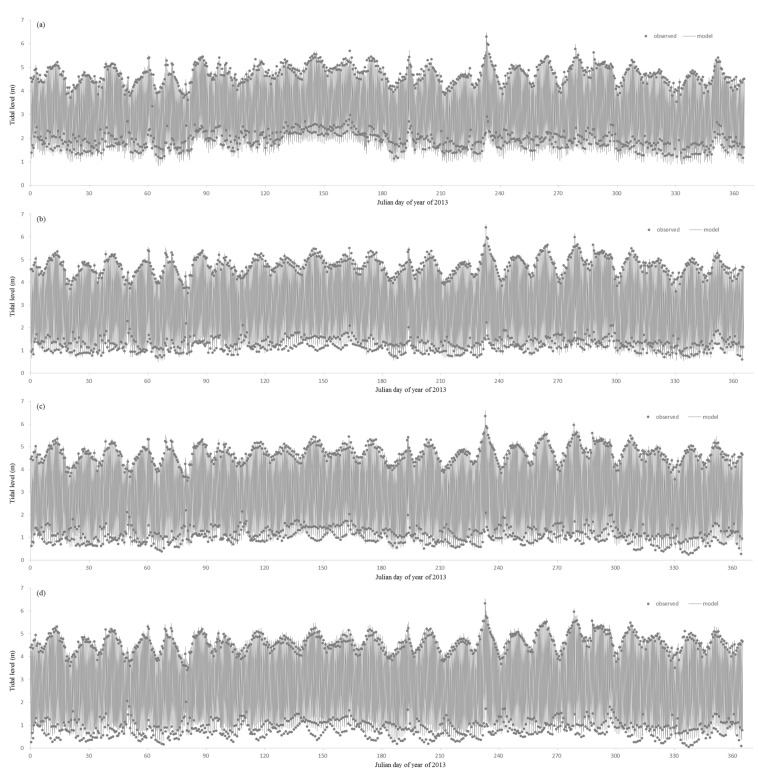
Tidal level calibration results for Wenshanli (**a**), Jiefang Bridge (**b**), Xianan (**c**) and Baiyantan (**d**).

**Figure 4:**
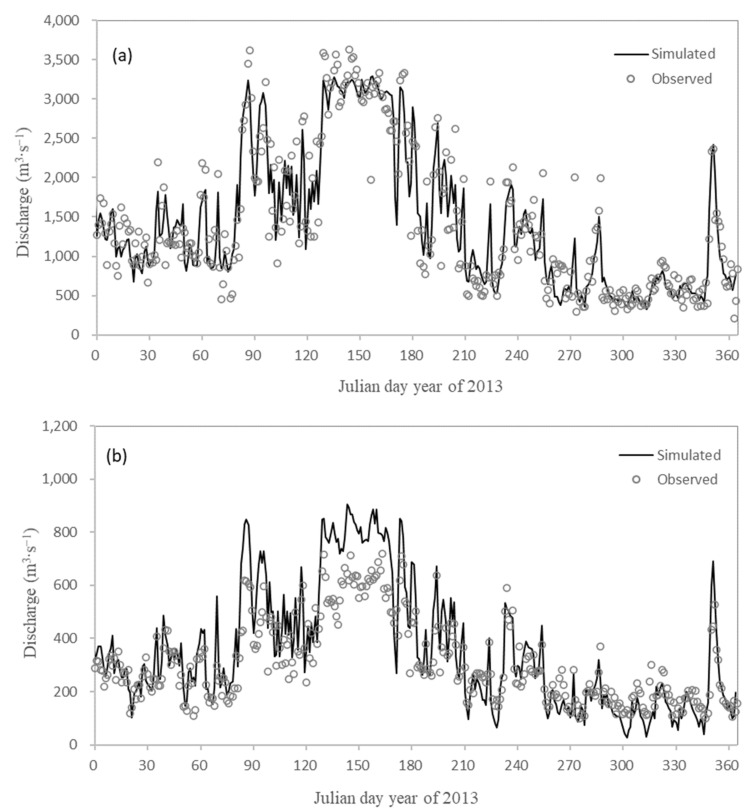
Discharge calibration results of Zhuqi (**a**) and Wenshanli (**b**).

The bottom roughness coefficient and wind field parameters were calibrated and verified through the field monitoring values of model-related parameters and relevant references. The final roughness parameter was 0.015–0.03 m, the wind drag coefficient was 3 × 10^−3^, and the wind occlusion coefficient was 1. The hydrodynamic calibration mainly verified the tidal level, flow, temperature, and salinity. The measured data of hydrological stations and tidal stations from January to December 2012 were used for model parameter calibration, and then the measured data from January to December 2013 were used for model parameter verification. Minjiang hydrodynamic model tidal level verification diagram in 2013 (Wenshanli, Jiefang Bridge, Xianan, and Baiyantan: two high tidal levels and two low tidal levels per day) was shown in Figure 3 and Table 2. Compared with the measured value and model simulation value, the average error was less than 0.17 m, the average absolute error was less than 0.23 m, and the average absolute error was less than 0.20 m accounted for 50.60%. The results show that the simulated tidal level of each station was in good agreement with the measured value. Minjiang hydrodynamic model flow verification in 2013 (Zhuqi and Wenshanli) was shown in Figure 4. Compared with the measured value and model simulation value, the relative error of Zhuqi station flow was 14.86%, the relative error of the Wenshanli station flow was 24.95%. It could be seen that there was a certain error between the simulated flow and the measured value. The simulation value of the Wenshanli station in the North Channel was slightly larger in the flood period of the Minjiang River, mainly because the South Channel beach had a certain flood discharge capacity in the discharge period, while the model had some shortcomings in the shoreline generalization. The flow in other periods was in good agreement. The overall simulation error was within the acceptable range. Taking Zhuqi flow as upstream inflow, the diversion ratio of the North Channel was calculated. The measured annual average value of diversion ratio of the North Channel was 24.69%, while the simulated annual average value was 23.97%. The relative error was 28.46%. The hydrodynamic model could basically reflect the diversion status of the South Channel and the North Channel.

**Figure 5:**
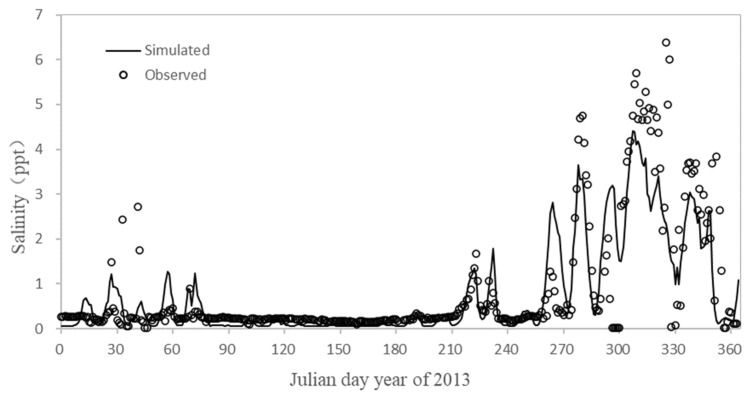
Salinity calibration results of Baiyantan.

The water temperature error of the hydrodynamic model of the Minjiang River in 2013 was shown in Table 3. Compared with the measured value and the simulated value, the average absolute error of all stations was 1.04 °C. The model accurately simulated the temporal and spatial distribution of the water temperature of the Minjiang River, indicating that the model could reasonably calculate the heat exchange process. Wenshanli and Zhuqi were not affected by saltwater. The salinity verification diagram of Baiyantan was shown in Figure 5. After removing individual points, the average relative error of salinity is 23.20%, the salinity simulation value of Baiyantan was basically consistent with the measured value.

##### Water Quality Model Parameters

The model was mainly calibrated and verified the water quality state variables, including DO, TN, TP, NH_4_^+^, and BOD_5_. The important water quality parameter values of the water quality module were shown in Table 1, mainly based on the calibration results, the previous field hydrological and water quality synchronous monitoring, and the research results of the Minjiang River related model [7] and other waters [32]. The error analysis of verification results of each monitoring point in the Minjiang River (Figure 1) was summarized in Table 3.

The relative error range of DO concentration (Table 3) at each monitoring station was 3.86% to 36.83%, of which Xiaxiyuan, Zhuqi, Kuiqi, and Min’an stations were within 17.00%. The absolute error between the average concentration of DO at the monitoring point and the simulated average concentration was within 0.50 mg L^−1^, indicating that the simulation results were good.

Due to the complex transport process of nutrient variables (TN, TP, and NH_4_^+^), the relative error was larger (Table 3). The average annual relative errors of TN, TP, NH_4_^+^, and BOD_5_ concentrations in all monitoring points were 10.30%, 16.25%, 30.50%, and 29.81%, respectively, and the results were within the acceptable range. Overall, the nutrient simulation results were in good agreement with the measured values, which basically reflected the spatial variation of nutrient deficiency in upstream water and poor water quality in the North Channel.

**Figure 6:**
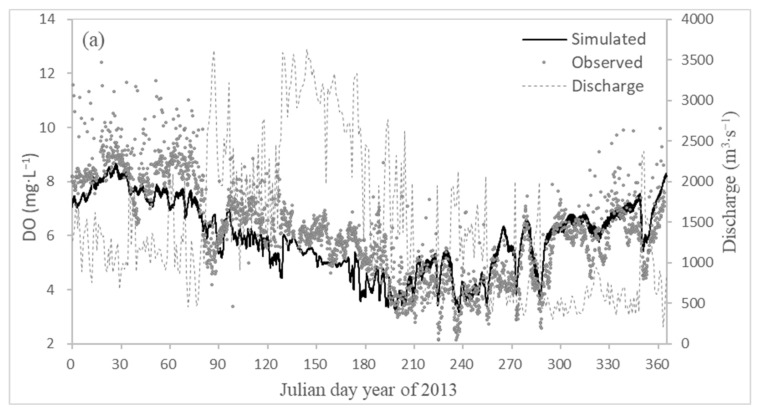

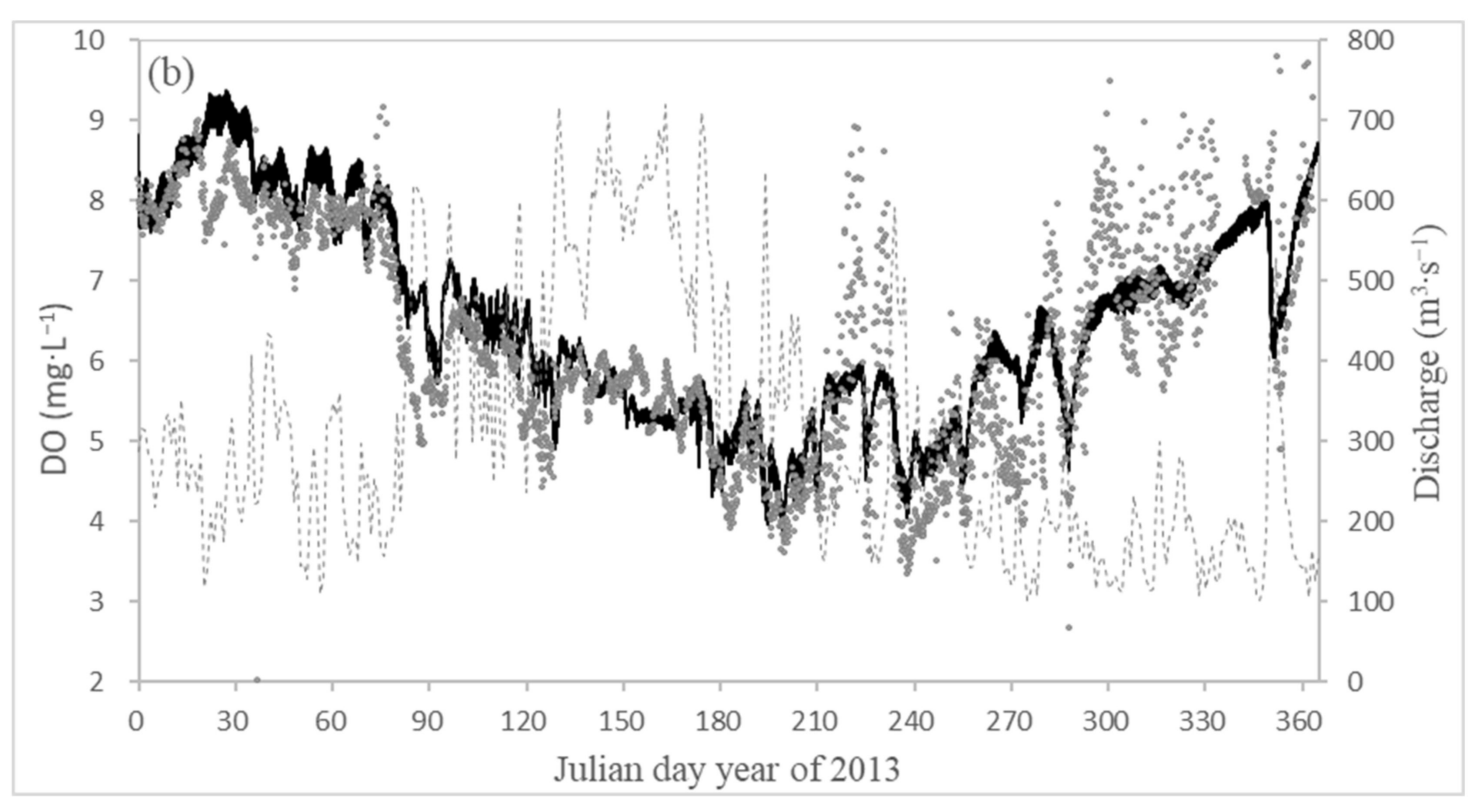
Relationship between simulated and measured DO values and flow and temperature changes in Zhuqi (**a**), Wenshanli (**b**) in 2013.

In order to further meet the research on the low oxygen problem in the downstream water body, the hourly frequency of DO calculated by the model was compared with the DO value of the automatic monitoring station, as shown in Figure 6. According to the DO simulation values of Zhuqi (a) and Wenshanli (b) in 2013, the comparison of the automatic monitoring station values with the flow and temperature (Figure 6), it could be seen that DO was significantly positively correlated with the runoff and was significantly negatively correlated with the temperature. The continuous hypoxia phenomenon mainly occurred in the period of high temperature and low flow. At the same time, the DO simulation value could also reflect the short-term hypoxia phenomenon in the downstream of the early flood discharge, such as October 13–19 (Julian day: 285–291), December 17–21 (Julian day: 350–354).

The verification results show that the established mathematical model of the Minjiang River water environment could better describe the hydrodynamic and temporal and spatial variation of water quality in the lower reaches of the Minjiang River and could fully reflect the real-time variation of DO in the Minjiang River.

## 3. Result and Discussion

### 3.1. Influence of Riverbed Incision on Downstream Water Age and Water Quality

**Figure 7:**
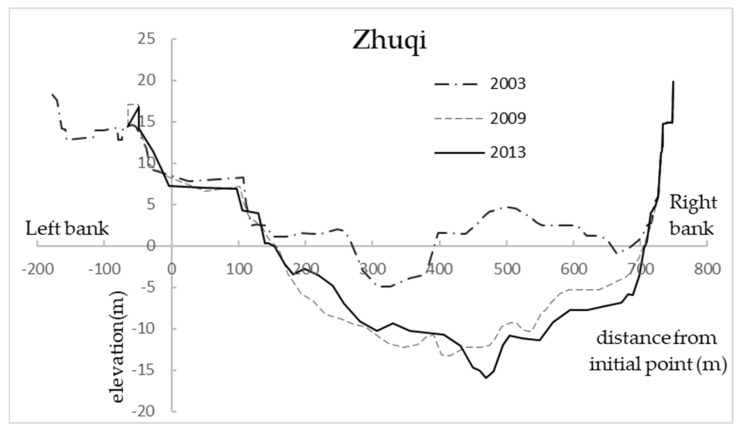
Topographic change of the cross section of Zhuqi hydrological station.

According to the analysis of the riverbed evolution of the lower reaches of the Minjiang River, since 1989, the riverbed incision in the lower reaches of the Minjiang River has been very serious, so it is necessary to study the influence of topographic changes caused by riverbed incision on downstream DO. The topographic change of typical hydrological station section (Zhuqi) was shown in Figure 7. The terrain of the Minjiang River in 2009 and 2003 was taken as the research object, and two calculation conditions were set up. As shown in Table 4, the terrain elevation in 2009 and 2003 was subtracted from the corresponding incision of four regions (Shuikou-Huai’an, North Channel, South Channel, end of the South Channel, and the North Channel-Changmen) on the basis of the terrain elevation in 2013. Other hydrological and meteorological conditions were present in 2013. According to the diversion ratios of the South Channel and the North Channel in 2009 and 2003, the rationality of the model calculation results under the terrain of each working condition was analyzed, and the local terrain of the model was adjusted accordingly. The analysis shows that the average diversion ratio of the North Channel in 2009 was 29%, the diversion ratio of the North Channel calculated by the model under D1 was about 27%. The average diversion ratio of the North Channel in 2003 was 81% (the average flow of Zhuqi station in 2003 was 1216 m^3^ s^−1^). When the discharge flow was less than 500 m^3^ s^−1^, the South Channel would be cut off, that is, the diversion ratio of the North Channel was more than 100%. The diversion ratio of the North Channel calculated by the model under D2 was about 74% (the average flow of Zhuqi station in 2003 was 1441 m^3^ s^−1^), and the South Channel would also be cut off when the flow was small. The error between the diversion ratio and the measured value of the North Channel was less than 30% under D1 and D2, indicating that the diversion ratios calculated by the model in 2009 and 2003 were basically consistent with the actual situation, which could reflect the actual diversion situation in 2009 and 2003.

**Figure 8:**
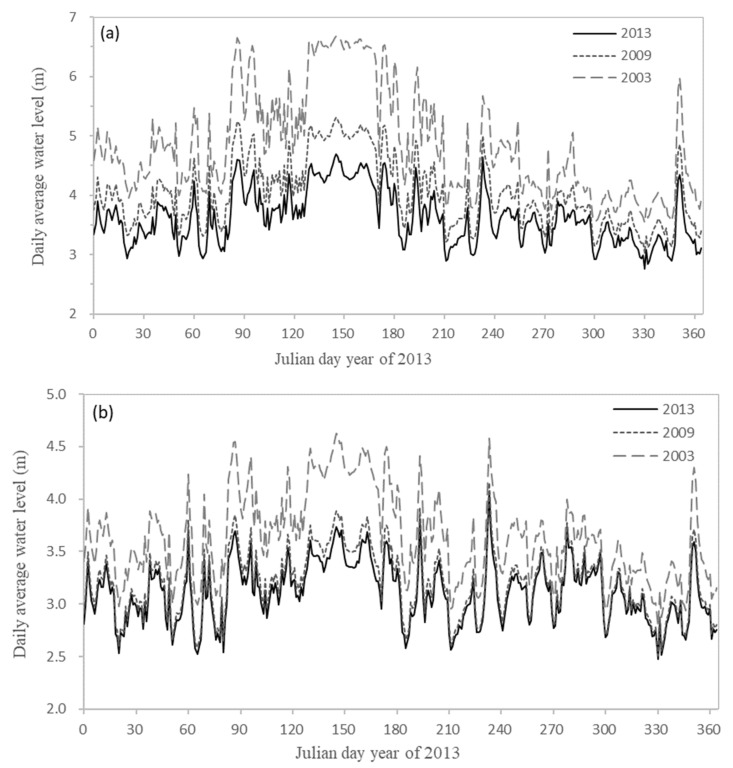
Water level of each section calculated under D1 and D2 ((**a**): Zhuqi; (**b**): Wenshanli) and current situation comparison chart.

Riverbed incision will lead to larger river volume and directly change the hydrodynamic conditions of the river, including tidal level and tidal current. According to the D1 and D2, the daily mean of water level (hourly frequency) and the daily mean of water age (hourly frequency) of two typical sections (Zhuqi: Figure 8a, Wenshanli: Figure 8b) in the lower reaches of the Minjiang River were calculated. The hydrodynamic results of the eight sections and the current situation were shown in Table 5. Geyangkou, Xiaxiyuan, and Zhuqi were mainly affected by runoff. Wenshanli and Kuiqi were in the North Channel; Wanbian was in the South Channel; Baiyantan and Min’an were the sections mainly affected by the tide. It could be seen from Figure 8 and Table 5 that the riverbed incision would lead to the overall decline of water level. The closer the river was to the downstream of Shuikou Reservoir, the greater the decline of water level would be. The decline of the tidal level near the outer sea was small, and the decline of water level from the upstream to the downstream was gradually reduced. For example, the annual average value of water level under D2 was gradually reduced from +4.28 m at Geyangkou to +0.34 m at Min’an, indicating that the lower reaches of the Minjiang River had gradually evolved from the river dominated by runoff to the river dominated by the tide. It also reflected the variation of the minimum water level of each section in the lower reaches of the Minjiang River, that is, the minimum water level of the section from Shuikou to Huai’an decreased significantly, and the variation range of the minimum water level of the section close to the offshore was small. The variation trend of water level in the year shows that the larger the discharge flow was, the larger the difference of the downstream water level under different calculated conditions would be (Figure 8). For example, when on 138 d (the runoff was about 3000 m^3^ s^−1^), the water levels of D1 and D2 increase by 0.71 m (average 0.41 m) and 2.30 m (average 1.23 m) compared with the current situation, while when on 264 d (the runoff was as low as 283 m^3^ s^−1^), the water levels of D1 and D2 increased by only 0.20 m and 0.47 m.

**Figure 9:**
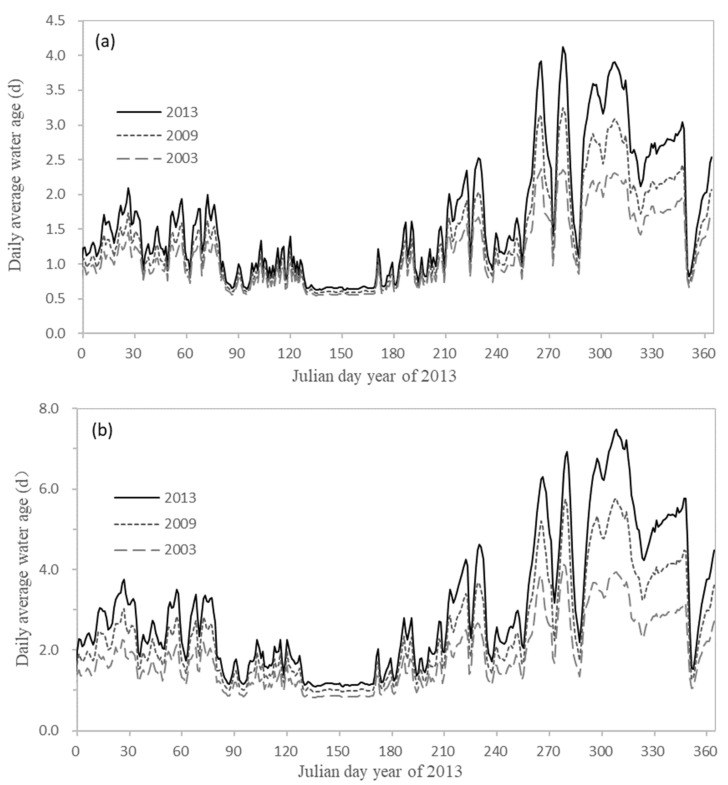
Water age of each section calculated under D1 and D2 ((**a**): Zhuqi; (**b**): Wenshanli) and current situation com-parison chart.

Before the riverbed incision, although the water level rose, the river volume was smaller than the current situation. The water age can show the time when the water flows to a specific position under the same flow and can indirectly reflect the volume of the river. The comparison of water ages of two typical sections in the lower reaches of the Minjiang River (Zhuqi: Figure 9a, Wenshanli Figure 9b) shows that the water ages of each section in the lower reaches of the Minjiang River under working conditions D1 and D2 were smaller than those of the current situation, indicating that the time for water to reach each section was faster under the same flow rate, and the river volume from Shuikou to the middle Minjing River was smaller. The biggest difference in the water age was the North Channel. Compared with the current situation of Kuiqi section under D1 and D2, the water age was reduced by 0.91 d and 2.55 d, respectively. The Geyangkou section is too close to the Shuikou Dam, and the reduction was not obvious. The influence of flood tide on the water age of Min’an section was also small, and the decrease is about 0.10 d under both conditions. Compared with the current situation, the water age of the Wanbian section of the South Channel was reduced by 0.72 d and 0.49 d under D1 and D2, respectively. It can be seen that the water age of the Wanbian section in 2003 was between 2009 and 2013, which was mainly due to the combined effect of the rise of river terrain and the sharp decrease of the diversion ratio of the South Channel. Although the terrain of the South Channel in 2003 was 1.82 m higher than that in 2009 with the smaller river volume, the diversion ratio of the South Channel in 2003 was only 25% (the diversion ratio of the South Channel in 2009 was 71%). The sharp decrease of upstream inflow led to the water age of the Wanbian section in 2003 was 0.23 d higher than that in 2009. Figure 9 also shows that the smaller the discharge was, the greater the difference between the calculated water age of each working condition would be, and the water level change was just the opposite. For example, when on 138 d (the runoff is about 3000 m^3^ s^−1^), the water age of working D1 and D2 was reduced by 0.05 d (average 0.27 d) and 0.08 d (average 0.49 d) compared with the current situation. When on 264 d (the runoff was about 283 m^3^ s^−1^, the water age of working D1 and D2 was reduced by 0.81 d and 1.58 d. It can be seen from the analysis that when the discharge is small, the water level under each working condition has a small increase compared with the current situation. The smaller river volume leads to a significant decrease in the downstream water age. When the discharge is large, the water level rises due to the hammed water of the river, and the smaller river volume caused by the higher terrain is compensated. Therefore, the smaller water age is not obvious. In addition, when the discharge is large, the water age of each section is small, resulting in a small difference between the water age of the downstream river and the current water age.

In recent decades, the deterioration of DO in the lower reaches of the Minjiang River has been closely related to the riverbed severe incision. The calculated results and the current situation of water quality of the eight sections in the lower reaches of the Minjiang River under working D1 and D2 are shown in Table 5. The DO of each section of Zhuqi, the North Channel, and the South Channel in the lower reaches of the Minjiang River under D1 and D2 increased compared with the current situation. The DO of the four sections under D1 increased by about 0.10 mg L^−1^, and the DO under D2 increased by 0.07–0.47 mg L^−1^, and the increase of the Wanbian section was the largest, which was 0.47 mg L^−1^.

NH_4_^+^ and BOD_5_ of each section calculated by D1 did not change significantly compared with the current situation. NH_4_^+^ and BOD_5_ of each section calculated by D2 changed differently compared with the current situation. The riverbed change from Shuikou to Huai’an was small. NH_4_^+^ and BOD_5_ of the Kuiqi section in the North Channel decreased by 0.22 mg·L^−1^ and 0.32 mg L^−1^, respectively. NH_4_^+^ and BOD_5_ of the Wanbian section in the South Channel increased by 0.10 mg L^−1^ and 0.24 mg L^−1^, respectively. NH_4_^+^ and BOD_5_ of Baiyantan and Min’an sections in the offshore channel of working D1 and D2 increased, but BOD_5_ decreased. The analysis showed that the diversion ratio of the North Channel was 74% on average under working D2, and the upstream water inflow increased. Under the same pollutant discharge, the water pollutants of the North Channel were diluted, and the concentration of each pollution index decreased. On the contrary, the water inflow of the South Channel decreased, and the concentration of water pollutants increased. This phenomenon was most obvious in the dry season. The changes of DO and water quality in the channel from Baiyantan to offshore were small, which were mainly affected by the tide of the sea. The DO in the rising tide of the sea was higher, while the organic carbon was higher, and NH_4_^+^ was lower. Therefore, in 2009 and 2003, the tidal fluctuation reduced at river terrain elevation, and the tidal advection diffusion ability was weakened, which led to the decrease of DO, DOC, and BOD_5_, and the increase of NH_4_^+^ in the channel from Baiyantan to offshore. Riverbed incision can also lead to the upward movement of the tidal zone boundary and tidal current boundary. The salinity of Baiyantan and Min’an section decreased by 0.13–0.33 ppt in 2003 and 2009 compared with the present situation (Table 5), which also proved that the sharp rise of tidal zone boundary and tidal current boundary in the lower reaches of the Minjiang River in recent decades was closely related to severe riverbed incision.

### 3.2. Influence of Tide and Sea Level Rise on Downstream Water Age and Water Quality

With global warming, high sea temperature, and melting of large glaciers eventually lead to sea-level rise [33,34,35]. The sea-level monitoring and analysis results in the latest China Sea Level Bulletin 2016 [36] show that the rate of sea-level rise along the coast of China was 3.2 mm a ^−1^ from 1980 to 2016, which was higher than the global average over the same period. In 2016, the sea-level along the coast of China was the highest since 1980, of which the sea level along the East China Sea was 115 mm higher than the normal year and 52 mm higher than in 2015. It was expected that the sea level along the East China Sea would rise 65–155 mm in the next 30 years. Due to the influence of the tide, the water exchange capacity of the estuary was enhanced, the water age was reduced, the water quality of the estuary was improved. In this study, under adverse conditions, the changes of water age and DO in the lower reaches of the Minjiang River with no tide and sea level rise were analyzed. Working D3 was no tidal change, that is, the water level of the outer boundary of the model (Meihua, Guantou) was the average water level of the outer sea (Table 6). The mid-term (in 2050) and long-term (in 2100) sea level rise of 150 mm and 430 mm scenarios were selected to simulate the changes of downstream water quality and water age, such as the D4 and D5 in Table 6.

Under D3, when there was no tidal effect, since the open boundary of the outer sea was the average water level of the outer sea, the water level of the lower reaches of the Minjiang River was reduced by 0.24–0.90 m. The flow velocity from Shuikou to the Zhuqi section becomes larger. The water age of the Zhuqi section decreased from 1.64 d to 1.42 d, and the DO of the Zhuqi section increased by 0.07 mg L^−1^. Without the influence of the tide, the water quality of the North Channel obviously deteriorated, among which the BOD_5_, DOC, NH_4_^+^, TN, and TP of the Kuiqi section increased (Table 7). The overall water depth of the North Channel decreased by 0.64 m, the flow velocity decreased by 0.34 m s^−1^, and the DO of the Kuiqi section decreased by 0.92 mg L^−1^. Under D3, the water quality concentration at the Wanbian section of the South Channel slightly decreased, which might be due to the fact that the diversion ratio of the South Channel increased by 0.6% (the average water volume increased by 9 m^3^ s^−1^) compared with the current condition, and the dilution water volume increased. The overall water depth of the South Channel decreased by 0.74 m, the flow velocity decreased by 0.25 m·s^−1^, and the DO at the Wanbian section was basically unchanged. The river channel from Baiyantan to the offshore was significantly affected by the tide. Under the action of no tide, the river channel was affected by the upward tracing of the salt tide, and the water depth was reduced by 0.40 m. After no tide, the overall flow rate was reduced by 0.64 m s^−1^. Due to the diffusion and dilution of oxygen-rich seawater offshore, the DO of Baiyantan and Min’an sections was reduced by 0.7 mg·L^−1^ and 1.02 mg L^−1^, respectively. Due to the high organic carbon in the offshore and the low NH_4_^+^, the DOC and BOD_5_ of the water body from Baiyantan to the offshore were reduced after no tide, and NH_4_^+^, TN, and TP were increased.

With the sea levels rise by 0.15 m and 0.43 m under D4 and D5, respectively, due to the enhancement of tidal effect, the water level rose in the lower reaches of the Minjiang River. Under the two conditions, the average water level in the lower reaches rose by 0.12 m and 0.35 m, respectively, and the volume of the upper reaches of the river increased, and the water age decreased.

When the tide rose, due to the enhanced tidal diffusion capacity, increased tidal volume, enhanced salinity intrusion, the water age decreased, DO increased, and DOC and BOD_5_ decreased, and NH_4_^+^, TN, and TP increased in the channel from Min’an to the offshore. When the tide fell, the water age of the water body from Min’an to the offshore increased, the DO decreased, and the water quality was also relatively poor. Overall, DO in river waters near the offshore has improved. The research results once again prove that the water level, water age, and water quality of the lower reaches of the Minjiang River were affected by topography, runoff, tide, and pollutant discharge. The hydrological and water quality characteristics of the upper reaches were mainly controlled by topography, runoff, and pollutant discharge. The hydrological and water quality characteristics of the water close to the offshore were more affected by tidal current transport and pollutant discharge [37].

## 4. Conclusions

This paper was to investigate the response of water age and water quality to riverbed incision and hydrological evolution by a hydrodynamics and WQ model in the Minjiang River. The comparison between observed data and model results for the calibration period of the year 2013 demonstrated that the integrated hydrodynamic and WQ model represented the major features of hydrology and WQ distributions in the Minjiang River. The main conclusions are as follows:

(1) The riverbed incision in the lower reaches of the Minjiang River is 3 m from 2003 to 2013, which led to the increase of river volume and the increase of downstream water age. Due to the accumulation of pollutants, the water quality deteriorated in varying degrees from Shuikou to Baiyantan reach, especially DO decreased by more than 0.50 mg·L^−1^ in the South and North Channel. However, due to the decline of topography, the tidal volume of the estuary increased, the dilution effect of offshore oxygen-enriched water increased, and the DO of the water below Baiyantan increased. Therefore, it is necessary to prevent the continuous undercutting of the riverbed to avoid the continuous deterioration of DO and the invasion of the salt tide in the South and North Channel.
(2) Under the effect of no tide, the overall water depth of the North Channel decreases by 0.64 m, the flow velocity decreases by 0.34 m·s^−1^. Due to the large amount of domestic wastewater not connected to the sewer network in Fuzhou, the takeover rate in Fuzhou is less than 60%. The accumulation of pollutants in the North Channel could not be quickly discharged to the sea. The DO of the Kuiqi section decreases by 0.92 mg·L^−1^. After the enhancement of tidal action, the dilution of oxygen-enriched water from the offshore water increased, and the DO increased.
(3) The hydrological and water quality characteristics of the upper part of the lower reaches of the Minjiang River were mainly controlled by topography, runoff, and pollutant discharge. The hydrological and water quality characteristics of the water close to the offshore were more affected by tidal current transport and pollutant discharge. Increased river discharge or the enhancement of tidal action had an important effect on improving DO by renewing water in the hypoxia section with more oxygenate, decreasing water age, and diluting nutrient concentration.

## Figures and Tables

**Table 1:** Main water quality parameters of the Minjiang River model.

Parameter	Parameter Definition (Unit)	Parameter Values in This Paper
*K_RO_*	Reoxygenation rate constant	$K_{R}=3.933∙\frac{u^{0.25}}{H^{0.65}}∙1.024^{\left(T-20\right)}$
*KT_RO_*	Temperature Rate Constant of Reoxygenation	1.024
*K_DC_*	DOC minimum dissolution rate (1/day)	0.03
*K_DCalg_*	Constants related to respiration of algae biomass (d^−1^/(g∙m^3^))	0.2
*AANOX*	Ratio of denitrification rate to oxic respiration rate of DOC	0.5
*Nit_m_*	Maximum nitrification rate (g N/m^3^/day)	0.07
*KHNit_DO_*	Semi-saturation constant of nitrification of DO	0.5
*KHNit_N_*	Semi-saturation constant of nitrification of NH_4_^+^	0.5
*K_CD_*	COD degradation rate (per day)	0.15
*K_HCOD_*	Semi-saturation number of degraded oxygen of COD (mg/L O_2_)	1.5
*PM_c_*	Maximum growth rate of cyanobacteria (1/day)	2.0
*BM_c_*	Basic metabolic rate of cyanobacteria (1/day)	0.04
*AOCR*	Quality of DO consumed per unit mass of NH_4_^+^ (g O_2_ per g N)	4.33
*AONT*	Ratio of DO to carbon during respiration (g O_2_ per g C)	2.67
*SOD*	Sediment oxygen demand (mg O/m^2^/day)(SOD < 0 Oxygen consumption of sediment from water)	−57–2000

**Table 2:** Statistics of tidal level simulation error in the lower reaches of the Minjiang River in 2013.

Tidal Level Error	Wenshanli	Jiefang Bridge	Xianan	Baiyantan
Mean error	0.17	0.01	0.03	−0.04
Mean absolute error	0.23	0.17	0.15	0.19
Proportion of average absolute	50.60%	67.02%	72.08%	60.95%
error < 0.20 m				

**Table 3:** Statistical analysis of water quality simulation errors in 2013.

Monitoring Station		Geyangkou	Xiaxiyuan	Zhuqi	Kuiqi	Wanbian	Min’an
Water temperature (°C)	Obs. Mean	21.38	21.27	21.41	22.00	21.80	22.04
	Sim. Mean	21.74	21.76	21.81	21.97	21.77	21.75
	*RE* (%)	5.78%	5.30%	3.48%	6.74%	3.10%	4.55%
DO(mg·L^−1^)	Obs. Mean	5.40	6.14	6.90	6.46	5.24	7.63
	Sim. Mean	4.27	5.16	5.92	6.26	6.96	7.49
	*RE* (%)	26.75%	15.71%	16.90%	11.16%	36.83%	3.86%
TN(mg·L^−1^)	Obs. Mean	1.49	1.50	1.52	1.79	1.84	1.78
	Sim. Mean	1.41	1.44	1.46	1.94	1.98	1.74
	*RE* (%)	5.32%	13.42%	15.16%	12.35%	10.62%	4.94%
TP(mg·L^−1^)	Obs. Mean	0.068	0.101	0.104	0.122	0.075	0.083
	Sim. Mean	0.066	0.079	0.085	0.135	0.096	0.086
	*RE* (%)	3.77%	23.21%	20.54%	15.47%	29.63%	4.88%
NH_4_^+^(mg·L^−1^)	Obs. Mean	0.14	0.24	0.24	0.83	0.45	0.21
	Sim. Mean	0.15	0.18	0.20	0.65	0.37	0.27
	*RE* (%)	1.80%	39.63%	57.87%	26.83%	24.81%	32.05%
BOD_5_(mg·L^−1^)	Obs. Mean	1.23	1.53	1.35	3.10	2.77	2.92
	Sim. Mean	0.91	1.10	1.07	2.56	2.53	2.48
	*RE* (%)	25.65%	39.90%	35.99%	26.53%	33.23%	17.57%

Note: Obs. Mean represents the observation average, Sim. Mean represents the simulated average, Relative Error (*RE*) calculation method is as follows: $RE=\frac{\frac{1}{N}\sum_{n=1}^{N}\left|O^{n}-P^{n}\right|}{\bar{O}}\times 100\%$, *N* is the number of observed and predicted values, $O^{n}$ is the nth observed value, $P^{n}$ is the nth predicted value, $\bar{O}$ the average observed value.

**Table 4:** Calculation conditions of influence of terrain change on downstream DO.

Calculation Condition	Riverbed Incision Compared with the Current Situation (m)				Remark
	Shuikou-Huai’an	North Channel	South Channel	End of the South Channel and the North Channel-Changmen	
Current situation	-	-	-	-	Topography in 2013
D1	1.82	0.81	1.34	0.80	Topography in 2009
D2	4.53	1.66	3.16	3.08	Topography in 2003

**Table 5:** Calculation conditions of influence of terrain change on downstream DO.

Calculation Condition	Section	Water Level	Water Age	DO	BOD_5_	DOC	NH_4_^+^	TN	TP	Salinity
current situation (2013)	Geyangkou	5.16	0.15	4.27	0.43	0.42	0.15	1.41	0.066	0.06
	Xiaxiyuan	4.31	0.59	5.16	0.50	0.48	0.18	1.44	0.079	0.06
	Zhuqi	3.63	1.64	5.92	0.51	0.50	0.20	1.46	0.085	0.06
	Wenshanli	3.11	3.01	6.54	0.55	0.53	0.27	1.54	0.094	0.06
	Kuiqi	2.87	5.15	6.26	1.22	0.98	0.65	1.94	0.135	0.08
	Wanbian	3.05	3.97	7.06	0.59	0.58	0.29	1.63	0.092	0.06
	Baiyantan	2.79	6.07	7.18	0.88	0.86	0.38	1.80	0.098	0.78
	Min’an	2.74	3.77	7.49	1.22	1.20	0.27	1.74	0.086	2.78
D1 (2009)	Geyangkou	6.74	0.14	4.27	0.43	0.42	0.15	1.41	0.066	0.06
	Xiaxiyuan	5.12	0.50	5.22	0.50	0.48	0.18	1.44	0.079	0.06
	Zhuqi	4.03	1.36	6.02	0.51	0.50	0.20	1.46	0.085	0.06
	Wenshanli	3.20	2.42	6.62	0.56	0.53	0.27	1.53	0.095	0.06
	Kuiqi	2.92	4.24	6.35	1.22	0.97	0.64	1.91	0.135	0.07
	Wanbian	3.15	3.25	7.16	0.61	0.59	0.29	1.64	0.092	0.06
	Baiyantan	2.83	5.60	7.20	0.85	0.82	0.40	1.81	0.100	0.66
	Min’an	2.80	3.65	7.49	1.18	1.16	0.29	1.75	0.087	2.52
D2 (2003)	Geyangkou	9.44	0.14	4.27	0.43	0.42	0.15	1.41	0.066	0.06
	Xiaxiyuan	7.23	0.45	5.23	0.51	0.49	0.18	1.44	0.080	0.06
	Zhuqi	4.86	1.15	6.11	0.52	0.51	0.20	1.46	0.085	0.06
	Wenshanli	3.65	1.83	6.61	0.54	0.52	0.23	1.50	0.091	0.06
	Kuiqi	3.24	2.60	6.38	0.90	0.76	0.43	1.67	0.114	0.06
	Wanbian	3.29	3.48	7.53	0.83	0.72	0.39	1.89	0.098	0.06
	Baiyantan	3.10	4.60	7.02	0.81	0.76	0.40	1.79	0.101	0.58
	Min’an	3.08	3.67	7.32	1.06	1.04	0.32	1.75	0.091	2.46

Note: water level unit: m, water age unit: d, DO, BOD_5_, DOC, NH_4_^+^, TP, TN units: mg L^−1^, salinity unit: ppt.

**Table 6:** Calculation conditions of downstream DO affected by tidal variation.

Calculation Condition	Offshore Tide	Remark
D3	No tide	No tidal effect
D4	+0.15 m based on tide in 2013	Sea level in 2050
D5	+0.43 m based on tide in 2013	Sea level in 2100

**Table 7:** Hydrodynamic and water quality results of each section calculated by D3, D4 and D5.

Calculation Condition	Section	Water Level	Water Age	DO	BOD_5_	DOC	NH_4_^+^	TN	TP	Salinity
D3	Geyangkou	4.92	0.14	4.27	0.91	0.42	0.15	1.41	0.066	0.06
	Xiaxiyuan	3.49	0.51	5.22	1.13	0.49	0.18	1.44	0.079	0.06
	Zhuqi	2.73	1.42	5.99	1.09	0.50	0.20	1.46	0.085	0.06
	Wenshanli	2.43	2.54	6.52	1.16	0.54	0.28	1.54	0.097	0.06
	Kuiqi	2.38	4.14	5.34	3.65	1.24	0.82	2.02	0.164	0.06
	Wanbian	2.41	3.46	7.06	1.03	0.52	0.26	1.54	0.091	0.06
	Baiyantan	2.38	6.80	6.48	1.52	0.73	0.45	1.85	0.106	0.06
	Min’an	2.37	7.73	6.47	1.48	0.81	0.38	1.83	0.099	0.06
D4	Geyangkou	5.21	0.15	4.27	0.91	0.42	0.15	1.41	0.066	0.06
	Xiaxiyuan	4.41	0.59	5.16	1.10	0.48	0.17	1.44	0.078	0.06
	Zhuqi	3.75	1.66	5.92	1.06	0.50	0.20	1.46	0.085	0.06
	Wenshanli	3.25	3.07	6.54	1.09	0.53	0.27	1.54	0.094	0.06
	Kuiqi	3.01	5.23	6.26	2.53	0.97	0.64	1.93	0.134	0.09
	Wanbian	3.18	4.05	7.06	1.25	0.58	0.29	1.63	0.092	0.06
	Baiyantan	2.93	6.09	7.19	1.75	0.87	0.38	1.79	0.098	0.80
	Min’an	2.88	3.75	7.50	2.48	1.21	0.27	1.74	0.085	2.81
D5	Geyangkou	5.31	0.15	4.27	0.91	0.42	0.15	1.41	0.066	0.06
	Xiaxiyuan	4.60	0.61	5.17	1.09	0.48	0.17	1.44	0.078	0.06
	Zhuqi	3.97	1.72	5.92	1.06	0.50	0.20	1.46	0.085	0.06
	Wenshanli	3.50	3.17	6.54	1.08	0.53	0.27	1.54	0.094	0.06
	Kuiqi	3.28	5.37	6.26	2.48	0.96	0.63	1.93	0.133	0.09
	Wanbian	3.44	4.19	7.06	1.24	0.58	0.28	1.63	0.091	0.06
	Baiyantan	3.19	6.11	7.19	1.78	0.89	0.37	1.79	0.097	0.83
	Min’an	3.15	3.73	7.50	2.50	1.23	0.27	1.74	0.085	2.85

Note: water level unit: m, water age unit: d, DO, BOD_5_, DOC, NH_4_^+^, TP, TN units: mg·L^−1^, salinity unit: ppt.
